# Miscellany

**Published:** 1888-06

**Authors:** 


### MISCELLANY.

---

### A PROTEST AGAINST THE INTRODUCTION OF WATER GAS INTO PHILADELPHIA.

The College of Physicians of Philadelphia last week adopted a protest against the introduction of water gas into Philadelphia, based upon a report prepared by a special committee.

The committee reported that the various analyses of water gas, as published, show that it contains from 30 to 45 per centum, or even more, of carbonic oxide, while ordinary coal gas has in it from 3 to 7 per centum of the same compound. A comparison of the deleterious or poisonous substances yielded to the surrounding atmosphere during the combustion of an equal volume of each of the gases showed that the combustion of 100 cubic feet of coal gas would give rise to 55.84 cubic feet of carbonic oxide gas; whilst the combustion of an equal volume of water gas would generate 73.32 cubic feet of carbonic oxide. That is, the combustion of a given volume of water gas would produce over 30 per cent. more carbonic oxide than the combustion of an equal volume of coal gas, and would, therefore contaminate about one-third more the air of a room.

The relative poisonous properties of the two gases before they are burnt, the report says, depends upon the quantity of carbonic oxide in them, the other ingredients being comparatively harmless.

Dangers that already exist from this source would be alarmingly multiplied by the substitution of water gas for coal gas. In Troy, N. Y., during the winter of 1886-87, over fifteen persons were overcome by the escape of water gas from a leak in the soil, and required medical attendance to revive them. The inhabitants of an entire row of houses in the same city were similarly overcome, and in the cases which terminated fatally death was attributed to carbonic oxide poisoning.

It has been ascertained from Mayor Fitler that sufficient water gas would be introduced into the gasometers in this city to constitute one third of the entire bulk used, and that the manufacturers who will bid for the contract to supply the water product admit that it will contain 30 per cent. of carbonic oxide. After it is mixed with the coal gas, the substance which is supplied for illuminating purposes will contain 30½ per cent. of carbonic oxide. There is no limit of safety in the percentage of carbonic oxide, the report says, and the line must be drawn arbitrarily. The committee believes that 10 per cent. ought to be the maximum in any gas intended for use by the public as an illuminant. In Massachusetts a protest against the use of water gas containing more than 10 per cent. of the deadly vapor was sent to the Legisiature with the signatures of all the leading physicians in the State, including the professors of Harvard College, and the medical examiners and health offlcers of the various counties and towns.

In conclusion, the committee recommended the adoption of the following, which was indorsed by the College of Physicians:

*Whereas*, the College of Physicians of Philadelphia has learned that it is proposed to furnish the citizens of Philadelphia with illuminating gas consisting of two parts of coal gas and one part of water gas, a mixture which would probably contain at least about 15 per cent. of carbonic oxide; and

*Whereas*, in the opinion of this college, the use of any illuminating gas which contains more than 10 per cent. of carbonic oxide would be attended with very grave risk to the health and lives of the citizens of Philadelphia; therefore, be it

*Resolved*, That the mayor of Philadelphia is hereby petitioned, that if, in his opinion, it be necessary to use water gas in connection with illuminating gas, that great care be taken to render it certain that the mixture shall at no time contain more than 10 per cent. of carbonic oxide.

---

### THE STATE MEDICAL SOCIETY OF ARKANSAS.

OFFICE OF THE SECRETARY,
*Little Rock*, *May* 15, 1888.

*Editor Chicago Journal and Examiner:*

DEAR SIR: In compliance with instructions I transmit herewith the following resolutions adopted at the Thirteenth Annual Session of the State Medical Society of Arkansas, held at Fort Smith, April 25, 26, and 27, 1888, and ordered to be furnished to the American Medical Association, the medical and religious press, and to the State medical societies, soliciting their co-operation in bringing about a correction of these grevious and palpable errors:

*Resolved*, That the members of the State Medical Society of Arkansas have for years observed with pain and mortification the patronage given to charlatanism in all its multifarious aspects by the religious press of our country.

*Resolved*, *further*, *and most specifically*, That the appearance in religious papers, ostensibly published for the inculcation of truth and morality, of serious homilies on prayer and praise side by side with cures for consumption, cancer, Bright's disease, and other incurable ailments, to which an editorial indorsement is often given, as well as secret preparations under the cloak of remedies for disease, but really intended for purposes of fœticide and other immoral uses, largely tends to shake the confidence of the profession of medicine in the integrity and purpose of the managers and editors of such journals.

*Resolved*, *further*, That it has been the well-known custom of the profession to render services gratuitously to clergymen, which we do not regret, nor do we purpose to recall, yet we must assert that the frequent occurrence of indorsements and recomendations of the clergy of peripatetic doctors and advertising charlatans has in many instances been the only reward of our gratuitous services.

*Resolved*, *further*, That we are aware that the editors of religious newspapers admit the painful situation in which these advertisements place them, and attempt to excuse themselves by saying that it is necessary to take these advertisements in order to obtain means to conduct their papers; but in the language of orthodox theology we would say: "Put behind you that damnable doctrine that we must do evil that good may come."

*Resolved*, *further*, That, as a society, we declare that the continued perpetration of the above offenses by some of the clergy and religious press brings harm to the bodies of their constituency, and damages materially their influence upon the thinking class of the medical profession.

*Resolved*, That the Secretary be instructed to furnish copies of these resolutions to the religious and medical press of the United States, to the American Medical Association, and to the State medical societies, soliciting their co-operation in bringing about a correction of these grievous and palpable errors.

Very Respectfully,

L. P. GIBSON, M. D.,

*Secretary.*

---

The following act has been passed:

*Be it enacted by the Senate and House of Representatives of the United States of America in Congress assembled*, That the consent of Congress is hereby given to the city of Chicago, county of Cook, State of Illinois, to extend a tunnel, or inlet pipes, into Lake Michigan, so far as may be deemed necessary to insure a supply of pure water, and to erect a pier, or piers, and crib in the navigable waters of said Lake, for the making, preserving, and working of said aqueducts or pipes or tunnel, the plan and location thereof to be subject to the approval of the Secretary of War: *Provided*, That said said city shall furnish and maintain at its own expense, such beacon-lights or other signals on such piers or crib, as the Light-House Board shall prescribe.

Approved, May 9, 1888.

---

It is announced that THE COOK COUNTY DIRECTORY OF PHYSICIANS AND SURGEONS, which is to be published under the supervision and control of the Physicians' Collection Association, is nearly ready for press, and that it will be delivered during the month of July. The association has undertaken to present to the profession a work of merit and of value. It is stated that the book will be entirely reliable in its directory pages, besides possessing much additional information valuable for reference.

---

NINTH INTERNATIONAL MEDICAL CONGRESS.--The first two volumes of the Report of the Transactions of the Congress held in Washington in September, 1887, have already been issued. The remaining three volumes will be distributed during the summer. The volumes issued give full reports of the work of the first three sections of the Congress viz.: General Medicine, General Surgery, and Military and Naval Surgery and Medicine.

They are issued in good form and reflect credit on the publication committee.

---

THE RUSH MONUMENT FUND.—The American Medical Association is continuing its efforts, through its committee appointed for that purpose, to secure funds sufficient to erect a monument to the memory of Dr. Benjamin Rush, of whom a portrait appeared in the May number of this journal.

Medical-Director A. L. Gihon, of the United States Navy, is chairman of the committee and Professor George H. Rohe, of Baltimore, Maryland, is secretary.

---

In accordance with a resolution adopted by the Faculty of Medicine of the University of Pennsylvania, the first number of a medical monthly will be issued October 1, 1888, under the title "The University Medical Magazine. Edited under the auspices of the Alumni and Faculty of Medicine of The University of Pennsylvania."

---

BOARDS OF HEALTH IN OHIO.—By a new law in Ohio a board of health is to be established in every city or village containing more than 500 inhabitants.

---

Cincinnati has a crematory constructed with all modern improvements.

